# The role of high mobility group proteins in cellular senescence mechanisms

**DOI:** 10.3389/fragi.2024.1486281

**Published:** 2024-10-23

**Authors:** Jia Chen, Hongyu Li, Yongyin Huang, Qiang Tang

**Affiliations:** ^1^ Heilongjiang University of Chinese Medicine, Harbin, China; ^2^ Second Affiliated Hospital of Heilongjiang University of Chinese Medicine, Harbin, China

**Keywords:** hmgs, aging, senescence, SASP, histone

## Abstract

Aging is a universal physiological phenomenon, and chronic age-related diseases have become one of the leading causes of human mortality, accounting for nearly half of all deaths. Studies have shown that reducing the incidence of these diseases can not only extend lifespan but also promote healthy aging. In recent years, the potential role of non-histone high-mobility group proteins (HMGs) in the regulation of aging and lifespan has attracted widespread attention. HMGs play critical roles in cellular senescence and associated diseases through various pathways, encompassing multi-layered mechanisms involving protein interactions, molecular regulation, and chromatin dynamics. This review provides a comprehensive analysis of the interactions between HMG family proteins and senescence-associated secretory phenotype (SASP), chromatin structure, and histone modifications, offering a deeper exploration of the pivotal functions and impacts of HMGs in the aging process. Furthermore, we summarize recent findings on the contributions of HMG proteins to aging and age-related diseases. HMG proteins not only regulate senescence-associated inflammation through modulating the SASP but also influence genomic stability and cell fate decisions via interactions with chromatin and histones. Targeting HMG proteins holds great potential in delaying the progression of aging and its associated diseases. This review aims to provide a systematic overview of HMG proteins’ roles in aging and to lay a solid foundation for future anti-aging drug development and therapeutic strategies. With the advancing understanding of the mechanisms by which HMGs regulate aging, developing therapeutic interventions targeting HMGs may emerge as a promising approach to extending lifespan and enhancing healthspan.

## Introduction

Aging is an intrinsic physiological process, with nearly half of human deaths attributed to chronic aging-related diseases, including neurodegenerative disorders, cardiovascular diseases, and cancer (Partridge et al., 2018; Kubben and Misteli, 2017). Research indicates that reducing the incidence of these age-related diseases can extend lifespan and promote healthy aging. Understanding the mechanisms of aging and their effects on cells and their environment is crucial for promoting widespread healthy aging (Liang et al., 2024).

Cellular senescence is a widespread stress response that can occur at any stage of life, and its essence lies in the permanent cell cycle arrest of proliferating cells. As individuals age, the abundance of senescent cells increases significantly, becoming a key hallmark of aging and age-related diseases (Campisi, 2013; Campisi and D’adda Di Fagagna, 2007; Herbig et al., 2006). In a narrow sense, senescence refers to a heterogeneous phenotype involving multiple effector mechanisms. In addition to the shared feature of cell cycle inhibition, other characteristics include the senescence-associated secretory phenotype (SASP), changes in chromatin structure, altered sensitivity to cell death, and persistent DNA damage signaling. These features collectively influence the functional state of cells and the health of surrounding tissues, driving the aging process and contributing to age-related pathological changes (Olan et al., 2023; López-Otín et al., 2023). Eukaryotic cells, as intricate biological systems, exhibit close ties between the senescence process and genomic structural changes (Starkova et al., 2023; Chikhirzhina et al., 2021). Among these, the High Mobility Group Proteins (HMGs) family, composed of highly conserved non-histone proteins associated with chromatin, is abundantly present in the nuclei of eukaryotic cells. HMGs are pivotal in regulating gene expression, maintaining genomic stability, and facilitating the dynamic remodeling of chromatin architecture. Research has shown that HMGs are key players in the initiation and progression of cellular senescence, influencing chromatin remodeling, DNA repair, and gene transcription, which in turn drive senescence-associated phenotypic shifts (Papantonis, 2021). The HMG family is diverse, with members having distinct functions. For instance, HMGN proteins have been shown to enhance UV-induced DNA damage repair, whereas HMGA and HMGB proteins may inhibit specific DNA repair mechanisms, potentially leading to mutations and chromosomal instability, particularly in senescent cells (Reeves, 2015; Nanduri et al., 2020). This functional diversity underscores the varied roles of HMGs and highlights their significance in the regulation of aging and longevity.

## Basic structure of HMGs protein

HMGs are small non-histone chromatin proteins first identified by Ernest Johns and colleagues using polyacrylamide gel electrophoresis in calf thymus chromatin. HMGs are characterized by high mobility, low molecular weight (less than 30 kDa), and rapid electrophoretic mobility (Grosschedl et al., 1994; Goodwin and Johns, 1973; Reeves, 2010). HMGs play multiple roles in chromatin structure and function, acting as key “architectural factors.” They form a dynamic protein network between nucleosomes, continuously reshaping and reconfiguring their chromatin binding sites to regulate nucleosome and chromatin architecture while coordinating essential processes such as transcription, replication, and DNA repair. Moreover, their interactions with chromatin are modulated by various factors, including chemical modifications of proteins or their chromatin targets, as well as competition with other nuclear proteins for chromatin binding sites (Reeves, 2010; Gerlitz et al., 2009). HMGs exhibit age-dependent and tissue-specific variability. Compared to juvenile rats, aged rats show elevated HMG levels in the liver and lungs, reduced levels in the thymus, heart, brain, and kidneys, and stable levels in the spleen and testes. These age-dependent changes in HMG protein levels across tissues reflect the differential states of chromatin function, cellular growth, and activity during aging (Prasad and Thakur, 1990).

The HMG family is currently categorized into three subfamilies: HMGA (the HMG-AT-hook family), HMGB (the HMG-box family), and HMGN (the HMG-nucleosome binding family). HMGA proteins include HMGA1 (HMGA1a, HMGA1b, and HMGA1c) and HMGA2. Structurally, they contain three distinct conserved AT-hook domains and an acidic C-terminal tail (Cleynen and de Ven, 2008). The AT-hook modifies chromatin structure in an ATP-independent manner, binding to AT-rich sequences in double-stranded DNA to alter DNA structure and interacting with transcription factors to regulate transcription (Wang et al., 2022; Parisi et al., 2020a). HMGA proteins are highly expressed during embryonic development but are significantly downregulated in differentiated adult tissues. As “architectural transcription factors,” they lack direct transcriptional activation capability but modulate the expression of multiple genes by interacting with transcription factors and other nuclear proteins, either positively or negatively (Parisi et al., 2020a; Vignali and Marracci, 2020a). In senescent cells, HMGA proteins can influence the SASP by interacting with nuclear factor kappa-B (NF-κB) (Nacarelli et al., 2019). This regulatory mechanism also plays a significant role in other cellular processes, such as proliferation and differentiation. Studies have shown that HMGA2 inhibits the expression of p16^INK4a^ and p21, which are associated with cellular senescence, through the activation of the PI3K/Akt/mTOR/p70S6K signaling cascade, thereby reducing or reversing the senescence process (Yu et al., 2013; Bjedov and Rallis, 2020).

HMGB proteins consist of four subtypes, each containing two highly conserved HMG-box domains, HMG box A and B, along with an acidic C-terminal tail. Each HMG-box comprises three α-helices arranged in an L-shape, with the concave surface binding to the minor groove of DNA in a sequence-independent manner (Niu et al., 2020). While the movement and DNA-binding activities of box A and B are independent, tandem HMG-boxes enhance their DNA-binding capacity. The acidic tail, when in contact with the DNA-binding surfaces of the two HMG-boxes, reduces the affinity of HMGBs for double-stranded DNA but increases their affinity for distorted DNA. HMGB proteins function as “structural” chromatin proteins by inducing DNA bending and recognizing distorted DNA structures. Additionally, post-translational modifications and higher-order chromatin conformations affect their chromatin-binding capacity (Reeves, 2010; Gerlitz et al., 2009; Anggayasti et al., 2020). Although HMGB proteins lack sequence specificity, they significantly influence gene expression regulation by bending DNA and facilitating nucleosome sliding (Voong et al., 2021). Beyond their nuclear functions, HMGB1 can be passively released into the extracellular space in response to cellular stress or damage, acting as a damage-associated molecular pattern (DAMP) to regulate inflammation and immune responses (Sofiadis et al., 2021). Extracellularly, HMGB1 interacts with various receptors, functioning as a cytokine and chemokine to promote tissue repair and regeneration (Kang et al., 2014).

HMGN (HMG-14/17) proteins are unique to vertebrates and include five subtypes: HMGN1-5. Although high mobility group proteins resembling HMGN have been identified in some invertebrates, these proteins are typically classified within the HMGA or HMGB families rather than the HMGN family (Aleporou-Marinou et al., 2003; Claus et al., 1994). HMGNs contain three key functional regions: a bipartite nuclear localization signal, a nucleosome-binding domain (NBD) approximately 30 amino acids long, and an acidic chromatin unfolding domain (Reeves, 2010). Among these, the NBD specifically interacts with nucleosome core particles and contains a conserved eight-amino-acid motif, RRSARLSA, encoded by a single exon in all HMGNs. In living cells, HMGNs form a dynamic protein network within the nucleus, continually moving between nucleosomes to reshape and reconfigure their chromatin binding sites (Ueda et al., 2008). HMGN proteins play crucial roles in cellular stress responses and development. They regulate DNA repair processes, maintain genome stability, and, in some cases, act as “alarm proteins” involved in immune responses (Yang et al., 2018). Additionally, HMGN proteins influence the epigenetic regulation of histone modifications, thereby affecting the aging process (Nanduri et al., 2020).

## Effects of HMGs on SASP

The SASP refers to the phenomenon where senescent cells, while retaining metabolic activity, produce and secrete a variety of pro-inflammatory factors, chemokines, growth factors, proteases, and reactive oxygen species (Calcinotto et al., 2019; Coppe et al., 2010). SASP influences the behavior of neighboring cells via paracrine and autocrine signaling, altering the tissue microenvironment and accelerating aging. Due to its pro-inflammatory nature, prolonged SASP secretion can lead to a state of chronic low-grade inflammation, potentially shortening lifespan through inflammation-driven processes termed “inflammaging” (Salotti and Johnson, 2019).

HMGA proteins promote the formation of the SASP through various mechanisms, involving intricate signaling pathways and chromatin remodeling. By modulating metabolic pathways, altering chromatin structure, activating signaling cascades, and regulating microRNA expression, HMGA proteins play a significant role in SASP production and pro-inflammatory responses (Sgarra et al., 2020).

HMGA proteins regulate enhancer activity and upregulate the expression of nicotinamide phosphoribosyltransferase (NAMPT). NAMPT, a rate-limiting enzyme in the NAD + salvage pathway, enhances glycolysis and mitochondrial respiration, increasing the intracellular NAD+/NADH ratio, which subsequently inhibits AMP-activated protein kinase (AMPK) signaling. This suppression further reduces the inhibitory effect of p53 on the p38 MAPK pathway, leading to the activation of the p38 MAPK/NF-κB signaling cascade and the eventual upregulation of pro-inflammatory cytokines within SASP (Nacarelli et al., 2019; Salotti and Johnson, 2019; Hao et al., 2022). Notably, this process occurs independently of senescence-induced growth arrest, underscoring the critical role of HMGA in SASP regulation through metabolic pathways.

Moreover, SASP plays a crucial role in promoting the senescence phenotype in tumor cells. In certain cancer cells, HMGA1 can activate the PI3K/Akt signaling pathway and upregulate the expression of matrix metalloproteinase 9 (MMP-9) (Cheng et al., 2019). Specifically, HMGA1 promotes MMP-9 expression through PI3K/Akt activation, thereby enhancing cellular migration and invasion. While this mechanism is primarily studied in cancer cells, it may exert similar effects during SASP formation in senescent cells, facilitating the secretion of pro-inflammatory and tissue remodeling factors. Furthermore, HMGA1 regulates specific microRNAs, such as miR-181b, which influences cell proliferation and inflammatory responses. Research demonstrates that HMGA1 upregulates miR-181b expression, consequently promoting the expression of SASP-related genes (Moreno et al., 2023). MicroRNAs play a pivotal role in post-transcriptional regulation during cellular senescence, and HMGA’s modulation of microRNAs provides essential mechanistic support for SASP formation. p53, a key regulator of the senescence process, inhibits NF-κB activity through multiple mechanisms, thus reducing SASP production. First, p53 competes with NF-κB for the transcriptional coactivator p300/CBP, thereby suppressing NF-κB’s transcriptional activity. In addition, p53 inhibits NF-κB-inducing kinase (NIK) and upregulates the expression of the phosphatase WIP1, further suppressing NF-κB signaling activation (Jang et al., 2020). These mechanisms collectively reduce SASP formation, slowing the pro-inflammatory responses associated with aging. HMGA alters chromatin structure, enhancing MDM2-mediated ubiquitination of p53, promoting p53 degradation, and reducing its stability (Henningsen et al., 2021). As p53 levels decrease, its inhibitory effect on SASP is weakened, leading to an upregulation of SASP. This mechanism indirectly promotes SASP, indicating that HMGA regulates SASP formation not only directly but also through its impact on p53 stability.

Although the high expression of p16^INK4a^ is typically associated with cellular senescence, it does not directly lead to the production of SASP. Studies indicate that while p16^INK4a^ serves as a hallmark of senescence, it does not independently trigger SASP, which is instead regulated by DNA damage response pathways and other signaling mechanisms such as NF-κB and C/EBPβ (Safwan-Zaiter et al., 2022). In fact, p16INK4a can, to some extent, indirectly suppress the early onset of SASP by limiting the accumulation of DNA damage in cells (Buj et al., 2021). Research has demonstrated that in young stem cells, the high expression of HMGA2 aids in maintaining stem cell self-renewal capacity by inhibiting the expression of both p16^INK4a^ and p19Arf, thus preserving the proliferation and function of stem cells. Consequently, in early development and in younger cells, HMGA2 is regarded as a key regulatory factor in the suppression of aging (Kubota et al., 2021). As aging progresses, the expression of HMGA2 declines, which in turn leads to an increase in p16INK4a expression. Interestingly, although HMGA generally exhibits anti-senescent effects, it also indirectly contributes to the increase of SASP (Nacarelli et al., 2019).

HMGB1 is an RNA-binding protein (RBP) that, unlike HMGA, binds to hundreds of mRNAs, forming an RBP network that regulates senescence-related processes. By interacting with these mRNAs, HMGB1 modulates the availability of SASP-related transcripts, demonstrating its dual role in coordinating chromatin folding and RNA dynamics (Sofiadis et al., 2021). During cellular senescence, HMGB1 can be passively or actively released from the nucleus into the extracellular space, acting as a DAMP that exerts pro-inflammatory functions extracellularly. It activates pro-inflammatory factors within the SASP, thus amplifying intercellular inflammatory responses (Gaikwad and Kayed, 2022a). Additionally, HMGB1 promotes the secretion of key SASP factors such as IL-6 and MMP-3 by interacting with the receptor for advanced glycation end-products (RAGE) and the TLR-4 signaling pathway (García-Arnandis et al., 2010; Kim et al., 2018). Moreover, HMGB1 can directly bind to pro-inflammatory cytokines, such as IL-1β, IFN-γ, and TNF-α, synergistically enhancing their pro-inflammatory effects and accelerating SASP formation (Li et al., 2020). This synergism not only amplifies cytokine activity but also facilitates early activation and expansion of SASP by forming complexes. Studies have shown that when HMGB1 binds to IL-1β, it significantly enhances its pro-inflammatory action, driving the expansion of SASP (Cheng et al., 2017). Strategies aimed at depleting HMGB1 or using HMGB1-blocking antibodies effectively attenuate SASP secretion, further revealing HMGB1’s central role in senescence and establishing a link between HMGB1 redistribution, p53 activity, and senescence-associated inflammation. These findings provide potential therapeutic strategies for mitigating SASP (Zhang et al., 2016; Davalos et al., 2013).

Furthermore, upregulation of HMGB1 is associated not only with neuroinflammatory markers such as IL-6 and NF-κB, but also with pathological markers of brain aging. Pathological tau oligomers (TauO) promote HMGB1 release, triggering astrocyte senescence and enhancing the pro-inflammatory effects of SASP through paracrine signaling. Inhibiting HMGB1 expression improves cognitive function in aged and Alzheimer’s disease mouse models, highlighting the therapeutic potential of targeting HMGB1 in age-related cognitive decline (Gaikwad and Kayed, 2022b).

HMGB2 exhibits distinct pro-inflammatory mechanisms from HMGB1 during aging, particularly in its critical role in regulating SASP release. HMGB2 facilitates the production of SASP by promoting the recognition of cytoplasmic chromatin by cyclic GMP-AMP synthase (cGAS) (Zhao et al., 2020). As a cytosolic DNA sensor, cGAS non-specifically detects double-stranded DNA (dsDNA), playing a pivotal role in senescent cells and the formation of SASP (Hao et al., 2022; Takahashi et al., 2018). In senescent cells, the activation of cGAS primarily stems from cytoplasmic chromatin fragments (CCF) caused by nuclear membrane rupture, and cDNA produced by derepressed retrotransposons (Hao et al., 2022). HMGB2 plays a crucial auxiliary role in cGAS localization to CCF. Additionally, the topoisomerase 1-DNA covalent cleavage complex (TOP1cc), a vital component of CCF, is indispensable for cGAS-mediated cytoplasmic chromatin recognition and SASP production. The interaction between TOP1cc and cGAS enhances cGAS binding to dsDNA, triggering downstream signaling cascades that ultimately promote SASP release (Zhao et al., 2020).

**FIGURE 1:**
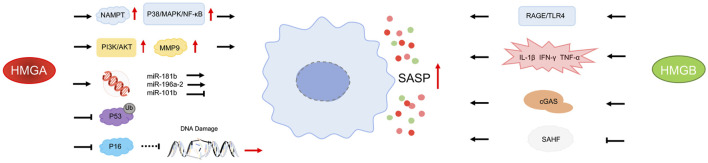
Effects of HMGs on SASP Arrows indicate promotion, blunt-headed arrows indicate inhibition, solid lines represent direct action, and dashed lines represent indirect action.

Another mechanism by which HMGB2 promotes SASP involves chromatin regulation. Although the overall levels of HMGB2 decline in senescent cells, its binding to chromatin significantly increases and plays a key regulatory role in the formation of senescence-associated heterochromatin foci (SAHF) (Guerrero and Gil, 2016). SAHF is a highly condensed heterochromatin structure formed in the nuclei of senescent cells, typically associated with gene silencing. However, HMGB2 prevents the spread of SAHF into SASP gene regions, maintaining the active expression of these genes. Studies have demonstrated that HMGB2 preferentially binds to SASP gene loci, such as those encoding C/EBP-β, IL-1β, IL-6, and IL-8, thereby preventing SAHF-mediated gene silencing (Aird et al., 2016). This mechanism ensures the sustained expression of SASP genes even in the context of widespread heterochromatin formation. Inhibiting HMGB2’s chromatin binding significantly reduces SASP gene expression without affecting cell cycle arrest, further illustrating the unique role of HMGB2 in regulating SASP (Figure 1) (Hao et al., 2022; Aird et al., 2016).

## HMGs and aging-associated chromatin structure

Factors that induce cellular senescence include epigenetic changes, telomere shortening, abnormal mitogenic signals, and genomic damage. The induction and maintenance of cell cycle arrest in senescence are primarily mediated through the p53/p21 and/or p16^INK4a^/pRB tumor suppressor pathways (Campisi, 2013; Gorgoulis et al., 2019). A hallmark of aging is the global alteration of chromatin structure, particularly the formation of SAHFs, which are multilayered structures centered around histone H3K9me3, Heterochromatin Protein 1(HP1) proteins, and macroH2A. The regulation of SAHFs involves both indirect effectors that promote the process and direct components physically associated with SAHFs, such as structural proteins and histone marks. SAHF formation involves a two-step process of heterochromatin mobilization and condensation, where p16^INK4a^ and its mediator RB play critical roles (Olan et al., 2023; Hao et al., 2022; Gorgoulis et al., 2019).

HMGA proteins, as chromatin architectural proteins, collaborate with p16^INK4a^ to promote the formation of SAHFs and the onset of senescence by regulating chromatin structure and limiting the accessibility of transcription factors. HMGA proteins stabilize the structure of SAHFs by modulating gene expression and preventing cells from escaping senescence (Parisi et al., 2020b). Moreover, HMGA proteins facilitate gene repression, particularly of proliferation-associated genes, through mechanisms involving alterations in DNA binding dynamics and the promotion of chromatin compaction. Heterochromatin is characterized by the accumulation of certain histone modifications, such as meK9H3, which generates docking sites for HP1. These biochemical changes lead to chromatin condensation and gene silencing (Narita et al., 2006; Postnikov and Bustin, 2016). HMGA proteins play a critical structural role in the formation of SAHFs, accumulating in senescent cells and binding to chromatin, thereby guiding and facilitating heterochromatin formation. In senescent cells, HMGA proteins localize to condensed DNA regions that also contain H3K9me3 and HP1, driving SAHF assembly and stabilizing cells in an irreversible state of senescence (Park et al., 2018; Li et al., 2023). Forced expression of HMGA proteins can trigger the formation of SAHFs, while inhibition of HMGA1 leads to the dissolution of SAHFs in senescent cells (Vignali and Marracci, 2020b) Another target of HMGA proteins is the retinoblastoma protein (Rb), with which they interact to inhibit E2F target genes during the cell cycle. This process is further reinforced through SAHF formation, ensuring that cells permanently exit the cell cycle during senescence (Schade et al., 2019; Shi et al., 2013).

Research has shown that overexpression of HMGA2 can induce SAHF formation and, in collaboration with the activation of p16INK4a and GSK3β, jointly promotes cell cycle inhibition and heterochromatin formation (Shi et al., 2017). Chemical agents that compete with HMGA proteins for binding to DNA minor grooves can decondense chromatin in senescent cells, suggesting that the biological activity of HMGA proteins in SAHF formation and senescence may be linked to the AT-hook domain of HMGA proteins (Reeves, 2010; Narita et al., 2006). The accumulation of HMGA proteins in senescent cells is associated with the loss of histone H1, which impacts chromatin structure and function. In conditions of induced premature senescence by ectopic expression of N-terminal EGFP-tagged histone H1, there is an accumulation of p16^INK4a^ and the formation of SAHFs, accompanied by a reduction in the endogenous histone H1 levels bound to chromatin. Co-expression of HMGA2 under these conditions results in a significant increase in SAHF formation, indicating that HMGA2 is necessary to replace histone H1 for SAHF formation (Funayama et al., 2006). HMGA proteins compete with histone H1 for linker DNA binding, thereby affecting chromatin compaction. In SAHFs, histone H1 is absent while HMGA1 and HMGA2 are enriched. It has been demonstrated that the incorporation of HMGA proteins into SAHFs is associated with the concurrent displacement of histone H1. Both HMGA proteins and histone H1 have the ability to significantly alter the structure of their binding substrates when interacting with double-stranded DNA or chromatin. However, HMGA proteins can outcompete histone H1 in binding to various DNA substrates, displacing histone H1 from the chromatin matrix (Hill et al., 1999; Hill and Reeves, 1997).

Sati et al. found that in oncogene-induced senescence (OIS), the weakened interaction between DNA methyltransferase 1 (DNMT1) and HMGA2 is crucial for SAHF formation (Sati et al., 2020). DNMT1 activates HMGA2 through DNA hypermethylation, promoting SAHF formation. As a transcriptional repressor, DNMT1 likely activates HMGA2 by suppressing the repressors of HMGA proteins, such as BRCA1 and ZNF350, through DNA hypermethylation, thereby inducing SAHF formation. HMGA2 competes with histone H1 to relieve chromatin compaction while maintaining long-range heterochromatin interactions (Sati et al., 2020). Senescent cells also exhibit a global increase in chromatin accessibility, although the genome-wide profile varies depending on the stimulus (Gorgoulis et al., 2019). In oncogenic RAS-induced senescent (RIS) cells, both autonomous and non-cell-autonomous activation of the NOTCH signaling pathway can inhibit SAHF formation and the development of RAS-driven accessible chromatin regions, in part through the suppression of HMGA1 transcription. Non-cell-autonomous activation of NOTCH1 signaling can suppress the formation of AT-rich, RAS-driven accessible regions at the nucleosome level and inhibit SAHF formation at the microscopic level (Parry et al., 2018).

Overall, the regulatory role of HMGA in SAHF formation promotes chromatin folding and the establishment of senescence. These changes in chromatin structure effectively block the regenerative potential of cells, thereby advancing the aging process. Inhibiting the expression of HMGA1 and HMGA2 significantly impacts the progression of cellular senescence, particularly by preventing SAHF formation, maintaining proliferative capacity, and delaying the onset of senescence. Studies have shown that inhibiting HMGA2 can slow down SAHF formation and reduce the accumulation of senescent cells, thereby combating age-related tissue functional decline (Shi et al., 2017; Yosef et al., 2016). Further experiments indicate that the knockout or downregulation of HMGA1 and HMGA2 not only reduces the number of SAHFs but also decreases SASP secretion, which has potential benefits for slowing the aging process (Shi et al., 2022). As a result, suppressing HMGA1 and HMGA2 expression may represent a promising therapeutic strategy for delaying aging and reducing age-related chronic diseases. However, further research is needed to confirm whether long-term inhibition of HMGA proteins may lead to other potential side effects, such as impaired tissue repair and regenerative capacity.

HMGB plays a crucial role in cellular senescence and the maintenance of genomic stability (Štros et al., 2007; Lu et al., 2019). One of the key mechanisms through which HMGB1 regulates cellular functions is its shuttling or translocation, which plays a significant role in cellular senescence. Unlike members of the HMGA protein family, HMGB1 shuttles between the nucleus and cytoplasm in a tightly regulated manner. HMGB1 can redistribute or relocate to the extracellular environment in senescent cells, where HMGB1 shuttling is considered a hallmark of senescent cells. This shuttling induces p53-dependent cellular senescence (Lu et al., 2019; Nacarelli et al., 2017). HMGB marks a subset of topologically associating domains (TADs) that contain genes essential for paracrine senescence, suggesting that as cells progress toward senescence, it plays a vital role in chromosomal spatial organization and gene expression regulation (Sofiadis et al., 2021).

HMGB proteins, mainly HMGB1 and HMGB2, influence SAHF formation through chromatin remodeling, gene regulation, and interactions with other chromatin regulators (Aird et al., 2016). The HMG-box domain within HMGB proteins enables them to specifically bind to bent, distorted, or damaged DNA regions, increasing chromatin plasticity and facilitating the binding of specific transcription factors to target genes (Štros, 2010; Kozlova et al., 2018; Štros et al., 2018). Additionally, HMGB proteins enhance gene accessibility by synergizing with chromatin remodeling complexes, such as SWI/SNF, promoting chromatin relaxation and nucleosome sliding. This process allows transcription factors and nucleosome remodeling complexes greater access to DNA, activating gene transcription, and guiding SAHF assembly during cellular senescence. This mechanism plays a critical role in the regulation of senescence-associated gene expression and the formation of the senescent phenotype (Štros, 2010; Sinha et al., 2017).

HMGB2 is a key regulatory factor involved in SAHF formation, exerting a similar role to that of HMGB1. In addition to preventing heterochromatin from spreading into SASP-related gene regions, as mentioned earlier, HMGB2 acts as an early primer in the onset of replicative senescence by influencing TAD gene expression. HMGB2 is associated with senescence in various cell types, driven by changes in genome structural organization. It maintains the inherent chromatin state of cells by forming looped chromatin structures, particularly during DNA replication, cell division, and the gradual restoration of three-dimensional chromatin architecture after cell division. HMGB2 can influence CTCF (CCCTC-binding factor), leading to the formation of looped structures in chromatin that maintain the 3D genomic conformation of proliferating cells, revealing a novel role for this looping factor (Zirkel et al., 2018a; Pugacheva et al., 2020). The absence of HMGB2 does not directly induce senescence, but its inhibition triggers three key features commonly seen in senescent cells: reduced transcriptional output, shifts in heterochromatin state, and the collapse of insulation at TAD boundaries bound by HMGB2. These changes may lead to a broad decline in cellular function (Starkova et al., 2023; Zirkel et al., 2018a). Additionally, the study found that as cells enter senescence, the three-dimensional organization of the genome suffers major disruption, due to the suppression of gene expression that maintains DNA conformation, and the noticeable depletion of HMGB2 before the appearance of typical senescence markers. These findings suggest that HMGB2-mediated relaxation of genome organization is a key event in triggering senescence programs across different cell lines, providing local insulation at TAD boundaries and within TADs, further highlighting its crucial role in maintaining genomic stability (Zirkel et al., 2018b).

## Competition between HMGs and histones

During cellular senescence, histone modifications play a crucial regulatory role by influencing chromatin structure and gene expression. Cellular aging is accompanied by widespread epigenetic changes, particularly alterations in histone acetylation and methylation. These proteins affect the aging process by modulating chromatin architecture. This relationship is primarily reflected in their competitive binding to DNA interaction sites and their regulation of chromatin structure (Gadecka and Bielak-Zmijewska, 2019).

HMGs and histones exhibit a competitive relationship at DNA binding sites. Histones bind to DNA in the form of octamers, forming the nucleosome, which is the basic unit of chromatin compaction. HMGs can specifically recognize and bind to DNA through domains such as the AT-hook and the C-terminal acidic tail, which often overlap with histone binding sites. Alternatively, HMGs can bind DNA simultaneously with histone H1 through a conserved eight-amino-acid sequence within the nucleosome-binding domain. When HMG proteins bind to these sites, they can induce nucleosome reorganization and chromatin relaxation, thus influencing gene accessibility and expression (Voong et al., 2021; Thomas and Stott, 2012). This state not only promotes active gene expression but also allows broader genomic reorganization, increasing responsiveness to environmental stimuli and aging signals (McCauley et al., 2018). Additionally, this alters the 3D structure of chromatin, leading to the activation of senescence-related genes, particularly pro-inflammatory genes, accelerating cellular aging signals and underlying the regulation of the aging process (Papantonis, 2021; Postnikov and Bustin, 2016; Amar et al., 2021). For example, during cellular aging or in response to external stimuli, the involvement of HMGs can lead to the activation of specific anti-aging or repair genes. Moreover, HMGs can enhance the activity of DNA ligase, thereby influencing DNA repair processes (Pellarin et al., 2016).

The acetylation and methylation levels of histones profoundly affect chromatin structure and gene expression, influencing cellular function and lifespan through their modification patterns (Cusack et al., 2020). HMGNs modulate chromatin structure both locally and globally by affecting post-translational modifications of histones, competing with histones H1/H5 for chromatin binding sites, and regulating the activity of chromatin remodeling factors. These actions, in turn, impact DNA-dependent processes such as transcription, recombination, and repair (Nizovtseva et al., 2019). Through these mechanisms, HMGNs induce chromatin fiber decompaction, increasing nucleosome DNA accessibility and activating genes, illustrating the close relationship between chromatin biological activity and its dynamic structure (Gerlitz et al., 2009; Gerlitz, 2010; Kim et al., 2008; Postnikov and Bustin, 2010). Although depletion of HMGNs does not significantly alter higher-order chromatin structures like TADs and A/B compartments, they mainly regulate chromatin states at the core structural level, exerting more localized effects without affecting overall 3D chromatin organization. Additionally, HMGNs influence cell-type-specific gene expression by modulating the accessibility of active chromatin regions (He et al., 2022).

The nucleosome binding sites of HMGNs partially overlap with those of linker histone H1, indicating that these two proteins can influence each other’s interactions with chromatin (Postnikov and Bustin, 2010). The nucleosome-binding domain (NBD) is a key determinant of HMGN-chromatin specificity, while the negatively charged C-terminal domain (RD) plays a crucial role in its regulatory functions. This domain determines the specificity of HMGN1 and HMGN2 for various histone modifications, while in mice, the C-terminus of HMGN5 targets the protein to euchromatin. It remains unclear whether this acidic region excludes HMGNs from binding to heterochromatin or promotes their interaction with nucleosomes in euchromatin. The C-terminus of HMGN1 enhances transcription in chromatin containing histone H1, whereas the C-terminus of HMGN5 directly interacts with the positively charged region of linker histone H5, mitigating the chromatin compaction induced by H5 (Nizovtseva et al., 2019). The interaction between HMGNs and linker histone H1 significantly influences the plasticity of chromatin fiber structure and function. Furthermore, studies have shown that HMGN5 not only colocalizes with CTCF in cells but may also open chromatin by displacing H1 at specific CTCF domains and shifting to nascent transcripts to regulate transcription (Araya et al., 2022).

This complex competitive relationship governs the expression patterns of key genes and serves as a critical mechanism for cells to adapt to physiological and environmental changes. A deeper understanding of these mechanisms holds significant theoretical importance for exploring gene regulatory networks during aging and developing related therapeutic strategies.

## HMGs and DNA damage

Aging is accompanied by the gradual accumulation of DNA damage due to endogenous metabolic processes like reactive oxygen species (ROS) production and external environmental factors such as ultraviolet radiation (Tiwari and Wilson, 2019). This damage, including double-strand breaks, base mismatches, and nucleotide modifications, compromises genomic stability. As the efficiency of cellular repair mechanisms declines, DNA damage becomes increasingly irreparable, leading to mutation accumulation, chromosomal aberrations, and cellular dysfunction, which ultimately accelerate tissue degeneration and organ failure (Kravvariti et al., 2023). The importance of DNA damage in aging is evident as it serves as a key driver of cellular senescence and a contributor to age-related diseases.

HMGB proteins are essential in limiting DNA damage and promoting DNA repair mechanisms, thereby preventing cellular senescence and delaying both aging and the onset of age-related diseases (Voong et al., 2021; Mukherjee and Vasquez, 2020). Age-related epigenetic changes, such as global hypomethylation and the reduction of Youth-DNA-gaps, are linked to the pathogenesis of DNA damage and age-related diseases. As cells age, the efflux of nuclear HMGB1 exacerbates the reduction of Youth-DNA-gaps, leading to increased DNA damage (Yasom et al., 2022). The Box A domain of HMGB1 functions as molecular scissors, creating Youth-DNA-gaps that maintain genomic stability by alleviating torsional stress in the DNA double helix and preventing structural damage to DNA (Yasom et al., 2022; Watcharanurak and Mutirangura, 2022). Additionally, the formation of Youth-DNA-gaps by Box A can prevent DNA double-strand breaks through histone deacetylation and SIRT1 activity, highlighting the potential of Youth-DNA-gaps as a novel strategy for monitoring and treating aging-related diseases (Watcharanurak and Mutirangura, 2022). These findings emphasize the importance of Youth-DNA-gap quantity as a biomarker for determining DNA aging stages, while the ability of HMGB1-generated Youth-DNA-gaps to reverse aging characteristics underscores HMGB1’s potential in maintaining genomic stability and preventing aging.

As a molecule integral to genomic stability, Box A-transfected cells demonstrate heightened expression of pluripotency markers OCT4, NANOG, and SOX2—key transcription factors governing stem cell pluripotency, self-renewal, and differentiation (Pitrone et al., 2017). The overexpression of these stemness markers suggests that Box A-transfected cells may possess anti-aging properties, potentially rejuvenating senescent cells by augmenting stem cell-like features as the induction of stemness increases (Ei et al., 2023). In addition to its role in nuclear DNA repair, HMGB proteins are directly involved in the maintenance of mitochondrial DNA integrity, playing a crucial role in mitochondrial DNA repair processes. Moreover, in the context of transgenic HMGB1 expression, the protein profoundly influences the phenotype and pathology of Atxn1 knockout mice, notably extending lifespan and improving motor function. Specifically, the mean lifespan of Atxn1 knockout mice increased from 217 days to 365.5 days (a 168% increase), and the maximum lifespan extended from 274 days to 448 days (a 163% increase) (Ito et al., 2014).

**FIGURE 2:**
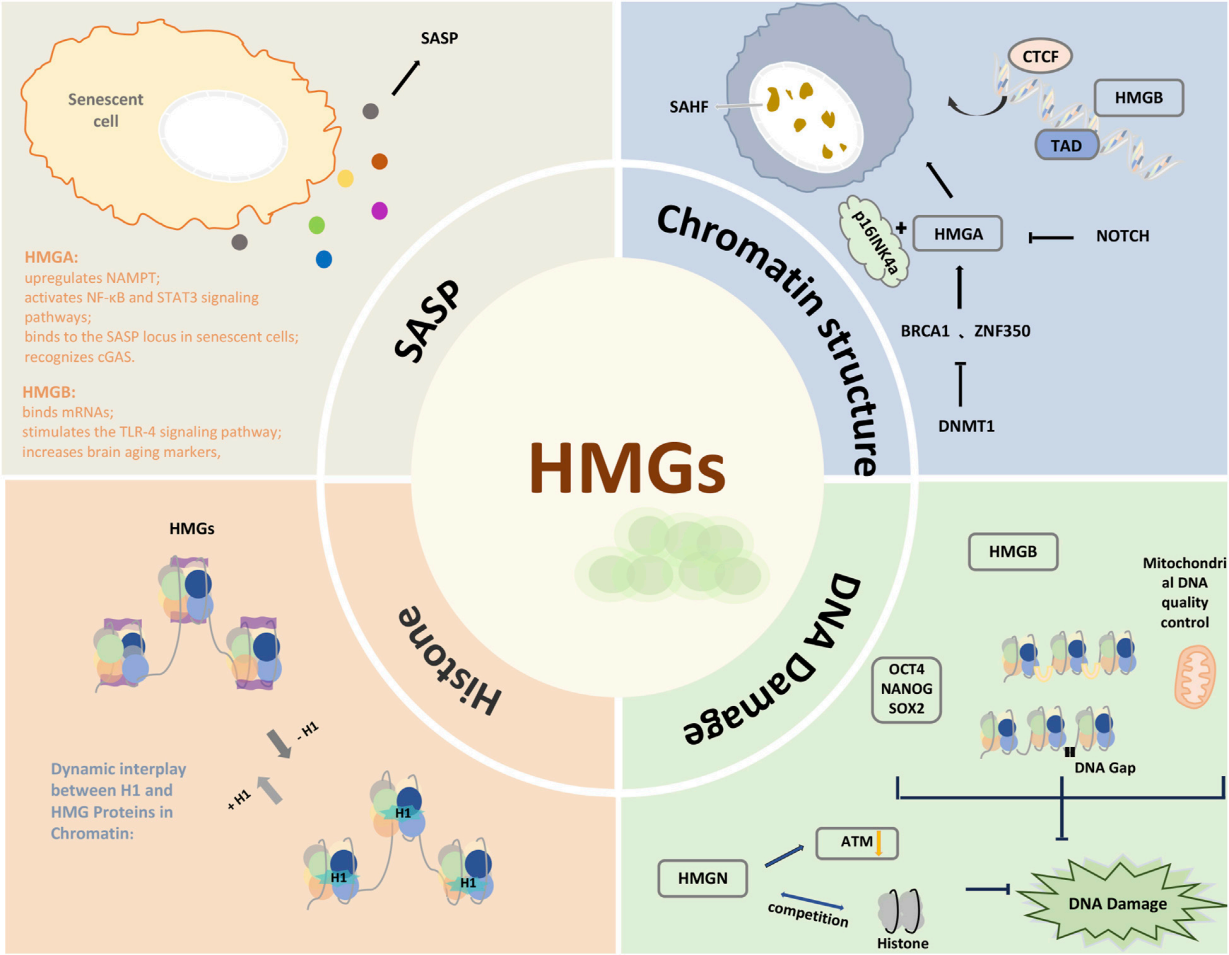
The diagrammatic summary of the mechanisms by which HMGs contribute to aging.

Although HMGNs are not DNA repair factors, they influence DNA repair processes due to their involvement in chromatin-based activities (Postnikov and Bustin, 2010). HMGNs are particularly important in regulating cellular responses to DNA damage induced by ultraviolet and ionizing radiation (Gerlitz, 2010; Kim et al., 2008). As chromatin structural proteins, sufficient levels of HMGN1 are required for the recruitment and activation of the key damage signal transducer ATM. HMGN1 modifies chromatin to influence the global organization of ATM throughout the nucleus, not just at double-strand break (DSB) sites. The absence of HMGN1 increases ATM retention in chromatin and decreases the relative phosphorylation level of ATM S1987p, thereby reducing ATM activation (Kim et al., 2008). Additionally, HMGNs compete with histone H1 for binding sites on chromatin, and the balance between their relative quantities and binding affinities regulates cellular responses to DNA damage. Histone H1 inhibits DNA repair processes, whereas HMGN1 activates DNA repair pathways by directly competing for histone H1 binding sites in chromatin and reducing H1’s inhibition of the HAT family (PCAF). After UV exposure, HMGN1 is recruited to damaged sites via transcription-coupled repair (TCR), where it removes histone H1 from the damaged regions or induces epigenetic changes that relax chromatin structure (Figure 2) (Gerlitz, 2010).

## HMG proteins in age-related diseases

### Neurodegenerative diseases

Neurodegenerative diseases are among the most prevalent chronic diseases globally, with incidence rates rapidly increasing with age. In these diseases, HMGB1, as a DAMP, plays a critical role in neurodegenerative conditions such as Alzheimer’s disease and Parkinson’s disease by activating inflammatory signaling pathways. The persistent inflammatory response not only exacerbates neuronal damage but also drives disease progression (Ikram et al., 2022). In Alzheimer’s disease, HMGB1 interacts with beta-amyloid (Aβ), promoting the formation of Aβ plaques and triggering axonal degradation through the TLR4-MARCKS signaling pathway, which is one of the hallmarks of early neurodegeneration. Studies have shown that therapies targeting HMGB1 have significant effects in Alzheimer’s disease models, reducing neurodegeneration and improving cognitive function (Paudel et al., 2020). Similarly, in Parkinson’s disease, HMGB1 is associated with the aggregation of α-synuclein, enhancing inflammatory signaling and further worsening neurodegeneration and motor function loss. Inhibition of HMGB1 has been shown to effectively reduce inflammation and alleviate disease symptoms (Guan et al., 2018). Furthermore, HMGB1 is implicated in the pathogenesis of other neurodegenerative diseases such as amyotrophic lateral sclerosis (ALS) and Huntington’s disease. Notably, the overexpression of HMGA family proteins may also accelerate neuronal death through abnormal cell cycle regulation. Research indicates that blocking HMGB1 release or inhibiting its signaling pathways could be a promising therapeutic strategy for these neurodegenerative diseases (Li et al., 2021; Fedele et al., 2001). Therefore, the role of HMGB1 in neurodegenerative diseases extends beyond its pro-inflammatory functions, contributing to the acceleration of neuronal degeneration and death. Targeting HMGB1 for therapeutic intervention presents significant clinical potential, offering a new avenue for the treatment of neurodegenerative diseases.

### Cardiovascular diseases

As age advances, damage to the cardiovascular system progressively accumulates, leading to a significant rise in the incidence of diseases such as atherosclerosis and myocardial infarction. The role of HMG proteins in cardiovascular diseases primarily involves regulating inflammatory responses, modulating gene expression, and participating in cellular damage repair mechanisms (Cai et al., 2020). The sterile inflammation triggered by the release of HMGB1 exacerbates damage to vascular endothelial cells and cardiomyocytes, driving disease progression. This inflammatory response is a key factor in the advancement of cardiovascular diseases such as atherosclerosis, myocardial infarction, and heart failure. Additionally, HMGB1 further aggravates cardiovascular pathology by regulating the inflammatory response during cardiac remodeling, myocardial hypertrophy, and ischemia-reperfusion injury (Andersson et al., 2018). HMGA2 also plays a significant role in cardiac development and myocardial remodeling. Studies have shown that HMGA2 regulates cardiac gene expression by interacting with cardiac transcription factors such as Nkx2.5, playing a crucial role in heart development and cardiomyocyte differentiation. During pressure overload-induced myocardial remodeling, upregulation of HMGA2 can alleviate cardiac dysfunction by activating the PPARγ/NRF2 signaling pathway, thereby exerting a cardioprotective effect (Wu et al., 2019). Therefore, regulating HMG proteins, particularly HMGB1 and HMGA2, could have a significant impact on the treatment of cardiovascular diseases. Blocking HMGB1’s inflammatory signaling pathways holds promise as a novel therapeutic strategy for cardiovascular diseases, while modulating HMGA2 expression has the potential to delay disease progression by improving myocardial remodeling and repair processes (Zhang et al., 2021). Overall, HMG proteins, through their multiple roles in inflammation, gene regulation, and tissue repair, have a profound influence on the occurrence and progression of cardiovascular diseases.

### Cancer

HMG proteins play a critical role in the initiation and progression of cancer, particularly in age-related cancers. HMGA proteins contribute to the formation and development of various cancers by altering chromatin structure and regulating gene expression. As chromatin regulators, HMGA proteins bind to specific DNA regions, influencing gene transcription and cell cycle regulation. In many malignancies, the overexpression of HMGA and HMGB proteins is closely linked to cancerous transformation, with their high levels often associated with poor prognosis (Cai et al., 2017). In age-related cancers such as lung cancer, breast cancer, colorectal cancer, and prostate cancer, elevated expression of HMGA1 and HMGA2 is correlated with increased self-renewal, invasiveness, and drug resistance in cancer cells (Giancotti et al., 2016). HMGA proteins also promote tumor proliferation, metastasis, and drug resistance through signaling pathways such as Wnt/β-catenin and PI3K/Akt. Additionally, HMGA proteins inhibit tumor suppressor genes like p53, reducing apoptosis and enhancing the survival capacity of cancer cells (Resar et al., 2018). Studies have shown that HMGA proteins further facilitate tumor progression by regulating the expression of microRNAs, such as the let-7 family (Busch et al., 2016).

Interestingly, high expression of HMGB1 is closely associated with immune evasion mechanisms in cancer, particularly in tumor-associated macrophages. HMGB1 increases the number of immunosuppressive cells, such as Tregs, thereby inhibiting anti-tumor immune responses (Wen and Zhang, 2023). Furthermore, although HMG proteins are generally involved in the aging process, this regulatory mechanism may, in some cases, help suppress tumor formation by preventing excessive proliferation and malignant transformation of cancer cells (Campisi, 2013; Zou et al., 2016).

However, anti-aging therapies may introduce new challenges, particularly by maintaining elevated levels of HMGA2, which could indirectly promote cancer development. Overexpression of HMGA2 is strongly associated with malignant transformation and tumor spread in various cancers (Maruyama et al., 2023). Therefore, while inhibiting aging can promote health and longevity in certain respects, its potential pro-cancer effects must be carefully evaluated to avoid increasing cancer risk (Campisi, 1997).

## Conclusion

In this review, we summarize the current research progress on non-histone high mobility group proteins (HMGs) in the field of aging, particularly their structural characteristics and functional roles in regulating the aging process. As chromatin architectural regulators, HMGs, in collaboration with histones, exert critical influence on chromatin dynamics and gene expression. By competitively binding to specific DNA sites, HMGs alter chromatin accessibility and regulate gene activity, thereby exerting profound effects at various stages of cellular senescence. This regulation involves a wide array of mechanisms and pathways, with a particularly notable impact on the senescence-associated secretory phenotype (SASP) and senescence-associated heterochromatin foci (SAHF).

HMG proteins, particularly members of the HMGA and HMGB families, play pivotal roles in the formation and regulation of SASP and SAHF. SASP comprises pro-inflammatory factors and proteins secreted during cellular senescence, which not only drive the aging process but are also closely associated with various age-related diseases, including chronic inflammation, cardiovascular diseases, and cancer. HMGA proteins promote or inhibit the spread of inflammatory signals by affecting chromatin structure and regulating the expression of SASP-related genes. Meanwhile, HMGB proteins, acting as damage-associated molecular patterns (DAMPs), activate inflammatory pathways and exacerbate the release of SASP. SAHF, as highly compacted heterochromatin regions, silence genes related to proliferation and the cell cycle, marking cells’ entry into a state of permanent arrest. The dynamic regulation of HMG proteins is crucial for the formation and maintenance of SAHF.

Recent studies have shown that targeting and blocking HMG proteins, particularly HMGB1 and HMGA2, not only reduces the release of SASP but also effectively inhibits inflammatory responses, thereby slowing the progression of age-related diseases (Gaikwad et al., 2021). By inhibiting the extracellular release of HMGB1, researchers have found that sterile inflammation and tissue damage can be alleviated, protecting cardiovascular health and delaying the development of age-related cardiovascular diseases. In metabolic diseases, HMGA1 regulates insulin signaling pathways, affecting insulin sensitivity and glucose metabolism homeostasis. Inhibiting HMGA1 expression could offer new strategies for treating type 2 diabetes. Thus, HMG proteins hold broad potential in maintaining metabolic homeostasis, controlling inflammation, regulating gene expression, and repairing tissue damage. Targeting these proteins has become a key direction in aging research. Compared to traditional therapies, targeting HMG proteins offers a more precise means of modulating age-related pathophysiological processes with fewer effects on normal cells. Additionally, considering the critical role of HMG proteins in many aging and age-related diseases, targeting HMG proteins could address multiple conditions while minimizing side effects (Kwon et al., 2020).

However, it is important to note that senescence is not solely a negative process. The dual nature of cellular senescence lies in its ability to both suppress the excessive proliferation of tumor cells and, conversely, promote the onset of age-related diseases through inflammation and tissue damage. Therefore, anti-aging strategies that target HMG proteins must be approached with caution, especially in contexts where tumor suppression mechanisms are compromised, as indiscriminate inhibition of senescence pathways could increase the risk of cancer. Thus, in developing anti-aging therapies targeting HMG proteins, it is crucial to balance the dual role of senescence in both tumor suppression and disease promotion. By conducting in-depth studies of the functions and mechanisms of HMG proteins in aging, we can gain a more comprehensive understanding of the molecular basis of cellular senescence, providing theoretical guidance and experimental evidence for developing more effective anti-aging interventions. Targeting HMG proteins not only offers the potential to delay aging but may also present new solutions for treating age-related diseases such as cancer, cardiovascular diseases, and metabolic disorders. Deep exploration in this area will not only help unravel the nature of cellular senescence but also lay a solid foundation for the future development of anti-aging drugs and therapeutic strategies.
